# High-Incidence Learning-Related Disabilities, Gender, and Educational and Employment Outcomes in Young Adulthood

**DOI:** 10.1177/00222194251340054

**Published:** 2025-05-20

**Authors:** Julia Stamp, Véronique Dupéré, Mathieu Pelletier-Dumas, Jiseul Sophia Ahn, Isabelle Plante, Isabelle Archambault

**Affiliations:** 1 Université de Montréal, Québec, Canada; 2 Université du Québec-Montréal, Canada

**Keywords:** high-incidence disabilities, learning disabilities, gender difference

## Abstract

The transition into post-secondary education or employment presents significant challenges for youth with high-incidence disabilities affecting learning, most commonly learning disabilities and attention-deficit/hyperactivity disorder. To date, few longitudinal studies investigate this transition in youth with learning-related disorders specifically, especially while considering education and employment outcomes simultaneously. This study examined relationships between learning-related disabilities requiring an individual intervention plan (Individualized Education Program [IEP]) in high school and key transition outcomes in early twenties in Quebec (*N* = 513; 61.4% with an IEP; 51.0% male). Compared with their normative peers, youth with learning-related disabilities were less likely to graduate from high school and enroll in college; more likely to be neither in education, employment, or training (NEET); and equally likely to be employed, regardless of the job type (career-related or not). Young women with disabilities were particularly likely to be NEET, and the gender gap in college enrollment favoring women narrowed among those with disabilities. Gender and disability status appear to intersect to shape critical early adulthood outcomes.

As adolescents reach the end of compulsory schooling, they must decide whether to continue in education, enter the workforce, or pursue other projects. For youth with high-incidence disabilities requiring Individualized Education Program (IEP), most commonly learning disabilities (LD) or attention-deficit/hyperactivity disorder (ADHD), this transition is particularly challenging (Etscheidt et al., 2023). For these students, even though post-secondary institutions are increasingly supportive, staying in education means entry into a much less regimented support system and continued exposure to risks of academic delays and failure (Chatoor, 2021). Conversely, rapid entry into the workforce also presents significant challenges: youth who do not pursue post-secondary education (especially those without a high school diploma) often struggle to find a stable, well-paying job corresponding to their aspirations and are at risk for chronic unemployment, poorer mental health, financial hardship, and social exclusion (Lavoie et al., 2021; Thouin et al., 2023).

With or without disabilities, the transition into satisfying education or employment is also shaped by contextual and sociodemographic factors affecting educational and employment expectations, aspirations, and opportunities, including gender (Flores et al., 2021; Haber et al., 2016; Mazzotti et al., 2021; Riegle-Crumb, 2019). Substantial gender gaps exist in both educational attainment and employment wherein continued education does not always materialize into more advantageous employment to the same degree across genders (Anderson et al., 2014; Jehn et al., 2021). Specifically, though young women attain higher levels of education than young men on average, they remain disadvantaged in the labor market in comparison to male counterparts, regardless of educational attainment, and these disparities appear emphasized for young women who do not continue into post-secondary education, a situation where many young women with learning-related disabilities find themselves (Lindsay, Cagliostro, & Carafa, 2018; Lindstrom et al., 2012).

Although educational and employment outcomes are closely interconnected for youth with learning-related disabilities, only a handful of recent studies adopt an integrated approach investigating these outcomes simultaneously (Cheatham & Randolph, 2022; Murray et al., 2021). Besides including gender as a control variable, these studies do not examine gender differences, despite these outcomes’ significant interconnectivity with gender. The goal of this study is thus to examine the relationship between learning-related high-incidence disabilities and a range of educational and employment outcomes for young people in transition, and whether these vary by gender.

## Transition Out of Compulsory Schooling and Into Education or Employment

The transition out of compulsory schooling is a significant developmental milestone for youth and involves important decisions, one such decision typically being whether to pursue continued education (e.g., college, vocational training, university) or transition into the workforce. In today’s competitive knowledge-based economy, exiting high school without a diploma, and to a lesser degree without entering post-secondary education, is associated with well-documented challenges in employment and other closely interconnected domains of functioning (e.g., health, social participation, housing; Lavoie et al., 2021). Consequently, youth remain in education longer on average than before. In Quebec, Canada, where the present study was conducted, the proportion of youth opting for post-secondary education has nearly doubled in the last two decades (Institut de la statistique du Québec, 2019).

### Youth With Learning-Related Disabilities

These trends toward higher educational expectations and attainment pose challenges for youth with high-incidence disabilities, a term used to denote disabilities found among students in the education system typically including LD, ADHD, mild intellectual disabilities, speech/language impairments, and emotional and behavioral disorders (Murray et al., 2021; Trainor et al., 2016). Among these, LD and ADHD are most common, accounting for about 60% of disabilities observed in this population in North America and often appearing comorbidly (Fletcher & Grigorenko, 2017; Horowitz et al., 2017; Moll et al., 2020; Statistics Canada, 2019). Although distinct neurodevelopmental disorders, LD and ADHD have important and overlapping impacts on educational functioning. Whereas LD typically impacts specific areas of learning (e.g., reading, writing, mathematics), ADHD affects executive functions critical in working correctly and efficiently (e.g., planning, problem solving, working memory), and both have widespread impacts on academic motivation and success (Espinet et al., 2022; Etscheidt et al., 2023; Fletcher & Grigorenko, 2017; Learning Disabilities Association of Canada, 2020; Murray et al., 2021). Life trajectories of youth with LD and/or ADHD are shaped by similar academic challenges and educational practices (e.g., Individualized Education Program [IEP], classroom accommodations), and both LD and ADHD are well-documented predictors of poorer educational and employment outcomes (Espinet et al., 2022; Etscheidt et al., 2023; Fletcher & Grigorenko, 2017; Murray et al., 2021). Given these commonalities, LD and ADHD are increasingly grouped together in educational practice and research (e.g., see Schimmele et al., 2021) and will be referred to together as learning-related disabilities throughout this paper.

The inclusion of students with disabilities in education is a legal right across North America, and measures are in place to protect this right (e.g., Ontario Ministry of Education [OME], 2022). In Canada, students with disabilities, including both LD and ADHD, are identified using government-issued disability categories, which, in the province of Quebec, are referred to with the French acronym “EHDAA,” denoting at-risk students and students with handicaps, social maladjustments or learning disabilities (*Élèves handicapés ou en difficulté d’adaptation ou d’apprentissage*). EHDAA categories are similar to the IDEA (Individuals with Disabilities Education Act) categories in the United States, with the exception that ADHD is included within EHDAA but not IDEA. As in the United States, students with special needs in Quebec are required to have an IEP, an official document produced by the school at both elementary and secondary levels that identifies specific learning needs and the means through which these will be addressed (i.e., accommodations; OME, 2022; Towle, 2015). Barring cognitive or physical impairments affecting learning (absence of which is a requirement for LD diagnosis), students with LD and/or ADHD with an IEP should, with appropriate support, be able to meet grade-level curriculum expectations and achieve the same success as their non-disabled peers and (OME, 2022).

### Learning-Related Disabilities, Compulsory Schooling, and Post-Secondary Education

Even with the implementation of IEPs, most students with high-incidence learning-related disabilities continue to experience academic delays and difficulties both during and out of compulsory schooling (Grigorenko et al., 2020). In 2007, a national report in Canada found that over a quarter of individuals ages 22 to 29 with LD had not obtained their high school diploma, a concerning figure in comparison to the national average which ranges from 5% to 15% (Homsy & Savard, 2018; Statistics Canada, 2019). For those who do succeed in obtaining their diploma, youth with disabilities remain consistently less likely to enroll in and complete post-secondary programs at all levels, often opting for shorter program leading to lesser qualifications, although their participation in post-secondary education is increasing (e.g., college, vocational training, university; Chatoor, 2021; Etscheidt et al., 2023).

Despite their increased participation in post-secondary education, many students with learning-related disabilities may elect not to enroll for reasons related to service provision. At the secondary school level, students who receive a formal diagnosis of learning-related disabilities are entitled to obtain an IEP (Towle, 2015). However, even with an IEP, receiving necessary supports depends on available services. Inadequate funding is the main obstacle in service provision, which unfortunately, means that students with disabilities who find themselves in public or lower SES schools are at even lesser chance of accessing services in a system where such services are already largely underfunded (Homsy & Savard, 2018; Towle, 2015). In that context, students with disabilities may doubt that the situation will improve should they enter post-secondary education and elect not to enroll at all. These students may expect difficulties in obtaining services, as post-secondary institutions in Canada are not required to provide the same breadth of accommodations that a student may have received in high school and these have no ready means of identifying students with disabilities unless they themselves register for services (Larose et al., 2022; Learning Disabilities Association of Ontario [LDAO], 2018).

### High-Incidence Disabilities and Participation in Employment

As an alternative to post-secondary education, many young people with high-incidence disabilities seek rapid entry into the workforce after high school (Murray et al., 2021). However, finding a job is a challenge for many people in that situation. In 2012, the employment rate of Canadians ages 15 to 24 with a learning-related disability was approximately half of that for those without any disability (25.9% vs. 51.9%), with the most common barrier to obtaining employment being inadequate training or experience (Arim, 2017). For those who do find employment, job situations are often not satisfactory as individuals with learning-related disabilities tend to work less hours and earn less than non-disabled peers, and must typically rely on unskilled, temporary, and minimum wage jobs (Arim, 2017; Rajah et al., 2023). Individuals with disabilities might decide not to seek employment or might accept jobs that do not correspond to their aspirations, due to fear of disclosing their disability or of not receiving accommodation, as services within employment, like in post-secondary education, rely on self-disclosure, and even among those who do, not all receive accommodations (Arim, 2017). Although government efforts to improve workplace practices are increasing, about half of young adults with disabilities still do not disclose needs to their employer, likely hindering employment stability and satisfaction (Arim, 2017; Employment and Social Development Canada, 2021).

## Gender and High-Incidence Disabilities: Impact on Education and Employment Outcomes

Learning disabilities, and similarly ADHD, are more commonly diagnosed among males than females (Mowlem et al., 2019). In Canada, the rate of LD among children 5 to 17 is 8.4% generally, and 10.6% among males versus only 6.1% among females (Statistics Canada, 2019; for similar statistics and gender differences with ADHD, see Espinet et al., 2022). Today, this discrepancy is generally considered to reflect a detection bias and contextual factors rather than biological underpinnings alone. For example, boys are more likely than girls to demonstrate externalizing behaviors (e.g., acting out, hyperactivity) alongside learning difficulties, facilitating their detection, whereas for girls, learning difficulties may need to be more pronounced to attract attention and be detected (Cunningham, n.d.; Hadad, 2020; Mowlem et al., 2019).

Even when detected, teachers tend to interpret girls’ difficulties as the result of being underachieving or disinterested in the subject matter more so than boys and are less likely to refer them for services (Auer et al., 2023; Little & McLennan, 2010; Quinn & Wagner, 2015). Consequently, girls are more likely to receive a late diagnosis during compulsory schooling, directly impacting access to an IEP and necessary services. This delayed recognition, and relative uncommonness of necessitating an IEP, may influence their self-perceptions (e.g., self-determination, self-efficacy) as they attempt to persevere through schooling alongside non-disabled peers, which are important factors in shaping post-secondary educational and employment choices (Burenkova et al., 2021; Lindstrom et al., 2020; Masdonati et al., 2022).

These gendered processes related to disabilities might modulate educational and employment gaps otherwise present in the general population. In terms of educational attainment, there are marked differences amongst young men and women at all levels. Namely, women typically demonstrate greater secondary and post-secondary educational attainment and are more likely to pursue collegial or university programs than men, who are more likely to enroll in training-based vocational programs (Anderson et al., 2014; Lindstrom et al., 2012). Women also tend to cite pursuing education by necessity (i.e., prerequisite for a desired career) more so than men, who more often terminate their studies by personal choice rather than obligation (Lindstrom et al., 2012; Parker, 2021). Despite these known differences, few studies investigate educational attainment by gender in reference to learning-related disabilities specifically. Nonetheless, large-scale survey research suggests that women’s academic advantage observed in the general population also applies to men and women with mild to moderate disabilities, though this may vary by the age group and disability type (Schimmele et al., 2021).

However, the academic advantage observed in women, with or without disabilities, does not translate to advantages in the workforce. While men typically attain lesser educational qualifications than women, they tend to report greater participation in employment and higher wages (Jehn et al., 2021). Among individuals with mild to moderate disabilities, women are twice as likely to work part time and more likely to occupy lower-wage jobs with fewer benefits and career advancement opportunities in comparison to their male counterparts (Morris et al., 2018; Schimmele et al., 2021). Young women with disabilities seeking rapid entry into the workforce out of compulsory schooling do appear to face truncated career choices, restricted by accumulated gender *and* disability barriers, although these trends may depend on the disability type (Lindsay, Cagliostro, & Carafa, 2018). Concretely, women are less likely to be exposed to and participate in vocational and training-based programs (often limited to gender-stereotyped service or childcare fields), and women with disabilities are likely to hold lesser occupational aspirations and less likely to participate in employment activities during schooling (Lindstrom et al., 2012).

## Gaps in Existing Literature

While preliminary research provides rationale that girls and boys identified with high-incidence learning-related disabilities likely face different psychological and social realities during their transition out of compulsory schooling, our understanding remains wanting due to sparse and often methodologically limited research. Tellingly, in a recent review of studies examining how gender intersected with disability in relation to employment outcomes among young adults, only five of the 48 reviewed studies focused on high-incidence learning-related disabilities, and of these, four relied on small samples (ranging between six and 129 participants, see Lindsay, Cagliostro, & Carafa, 2018). Overall, very few of the reviewed studies focused on disabilities of a specific type and severity and many grouped physical, intellectual, developmental, and learning-related disabilities together; these limitations likely obscure gender differences, given that the magnitude and direction of gender differences appear contingent on disability type. In addition to calling for more research focusing on specific types of disability in the field, the authors also highlighted the need to consider youth’s educational outcomes alongside employment outcomes, and insisted on the necessity, especially in quantitative studies, to consider not only whether youth were employed or not, but also how they perceived their employment situation. In short, there is a need for gender-informed studies that follow adolescent boys and girls with an IEP for high-incidence learning-related disabilities as they exit compulsory schooling regardless of whether they pursue employment *or* education, and that consider young people’s own appraisal of their employment situation (see also Hadad, 2020; Horowitz et al., 2017; Murray et al., 2021).

## Current Study

The goal of this present study is to examine how young men and women with high-incidence disabilities—notably LD and/or ADHD—requiring an IEP fare after exiting compulsory schooling in education and employment. Administrative data is used to identify the presence of an IEP for a learning-related disability (for which a diagnosis is necessary) during compulsory schooling, bypassing the limitations of less reliable self-report retrospective data. Participants with and without IEP during compulsory schooling were tracked regardless of whether they pursued education or employment years later. In addition, whereas much of the existing literature measures employment outcomes as a dichotomous variable (i.e., employed vs. not employed), this study nuances this by considering whether employment is perceived as temporary or within the desired field, a critical aspect for understanding school-to-work transition outcomes (Masdonati et al., 2022).

Besides gender, there are other internal and external factors that shape the school-to-work transition experiences of young adults with a disability. For example, Trainor and colleagues (2020) propose a research framework that places the individual with a disability at the core, interacting with outer “layers,” such as culture (e.g., familial factors, community types) and services (e.g., supports in education). To allow the investigation into potential gender differences while considering the intersectionality with other important sociodemographic factors, this study controlled for several background characteristics (see *Measures*). Although a detailed review is beyond the scope of this article, well-documented markers associated with elevated academic and employment challenges were incorporated as control variables, including visible minority status, parental education, and urban versus rural schooling (Dupéré et al., 2018; Lindsay et al., 2022).

It is hypothesized that when controlling for sociodemographic risk factors, disabilities will predict lower educational attainment as measured by (a) lesser likelihood of obtaining a high school diploma, and (b) lesser likelihood enrolling in post-secondary education. It is also hypothesized that disabilities will predict less favorable employment as measured by (c) greater likelihood of belonging neither in employment, education, or training (NEET), (d) greater likelihood of temporary employment, and (e) lesser likelihood of employment in a desired career field. Given current trends in educational attainment and employment observed in young men and women, we hypothesize that these relationships will vary as a function of gender, with women with disabilities facing greater challenges.

## Method

This study uses self-reported data collected as part of an ongoing longitudinal research project (for more information, see Dupéré et al., 2018). Data was also obtained via administrative records through the formal authorization on behalf of the *Commission d’accès à l’information* to access data from the Ministry of Education and Higher Education of Quebec, and school board records. The Institutional Review Board of the first author’s university approved the present study, and informed consent was obtained from the participants at all stages.

### General Sampling Procedures

Self-reported data was collected in two phases. Phase 1 was conducted between 2012 and 2015 in 12 socioeconomically disadvantaged French-language public high schools with high dropout rates located within metropolitan Montreal and surrounding areas. Students ages 14 to 18 from regular and some specialized classrooms (excluding classrooms for students with moderate to severe physical, mental, developmental, or intellectual disabilities) completed a self-report screening questionnaire assessing sociodemographic information and dropout risk (T1, *N* = 6,773; participation rate > 97%). Six months later (T2), on average, a subset of participants was invited to participate in an individual semi-structured interview (see *Measures*). This subset (*N* = 545; *M*_age_ = 16.3 years old [*SD* = 0.9]; 52% male; 31% having at least one parent born outside of Canada) was selected to include equal parts of (a) students having dropped out since T1, (b) risk-matched peers, and (c) normative peers. This procedure was designed to oversample youth with educational delays and interruptions, who represented two thirds of the sample (for details, see Dupéré et al., 2018). Phase 2 interviews were conducted between 2016 and 2020, as the participants reached their early 20s (T3, *n* = 386, *M*_age_ = 20.3 years old [*SD* = 0.9]). No significant sociodemographic differences were found between participants at T2 and T3 (Thouin et al., 2023).

Data from administrative records from the Ministry of Education were extracted in 2022 for the interview participants who did not opt out. Among the original sample of 545 participants, we excluded 32 participants, for whom administrative data was unavailable (*n* = 21) or those who had non-learning-related disabilities (*n* = 11; see Measures for details). This left an analytical sample of *n* = 513, with 61.4% of youth with IEP for learning-related disabilities and 51.0% male. Because youth at risk of high school dropout were oversampled and the participating schools had high scores on an official index of socioeconomic disadvantage used by the Ministry of Education (see Dupéré et al., 2018 for details), the proportion of participants with risk factors (e.g., with separated or divorced parents) was relatively high. Full descriptive statistics of the analytical sample are shown in Table 1 (see also Descriptive Results for details).

**Table 1.** Descriptives for Sociodemographic Variables, Educational Attainment, and Employment Outcomes.

Sociodemographic variables	Total %	No learning-related disability %	Learning-related disability status group %
	*N* = 513	*n* = 200^a^	*n* = 315^a^
Age	*M* = 16.31; *SD* = 0.91	*M* = 16.24; *SD* = 0.91	*M* = 16.36; *SD* = 0.91
Male	51.0	48.0	55.0
Lives with both parents	42.3	50.5	38.9
Parent born outside Canada^b^	35.6	35.4	37.0
Visible minority	23.3	22.7	25.1
Parental education^c^	55.8	53.3	58.8
Father employed full-time	76.0	85.9	72.0
Mother employed full-time	69.9	75.8	67.2
Urban school^d^	50.5	45.5	54.3
**Learning-related disability**	61.4		
**Educational attainment**	*N* = 513	*n* = 200^a^	*n* = 315^a^
High school diploma obtained	44.2	60.6	34.9
Enrolled in college since high school	30.8	42.9	23.8
**Employment outcomes**	(*n* = 362)	(*n* = 155)^a^	(*n* = 215)^a^
NEET^e^	14.9	9.7	18.6
Temporary job	32.7	32.9	32.6
Career-related job	18.9	19.4	18.6

*Note*. Age represents age at T1 (Phase 1). Learning-related disability status is based on *n* = 513 participants for which administrative data were available. Employment outcomes are based on *n* = 362 participants for whom data was available at T2 (Phase 2). NEET = neither in education, employment, or training.

a Frequencies have been rounded up to the next number based on 5 to protect confidentiality and rules for administrative data access and diffusion. ^b^At least one parent born outside of Canada. ^c^Highest parental education is high school diploma or less. ^d^School located in urban neighborhood. ^e^Not in Education, Employment, or Training at the time of the interview.

### Measures

#### Participant Interviews

Interviews were conducted in Phase 1 and Phase 2, between 2012–2015 and 2016–2020, respectively. The interviews included structured (i.e., precise questions about the job type, weekly working hours) and semi-structured portions (see Dupéré et al., 2018). The interviews lasted approximately 90 min, during which participants were asked to elaborate on different life spheres, including employment, education, health, and relationships. For the purpose of the present study, employment outcomes were obtained from the interview data, while demographics and educational variables were obtained from the initial survey data and administrative records.

#### High-Incidence Disability Status

A variable identifying students with high-incidence disabilities affecting learning (namely LD and ADHD) was created by combining two variables extracted from administrative data from Quebec’s Ministry of Education. A first variable identified students with an IEP in high school (for which a formal diagnosis is compulsory). A second identified the type of disability using the provincial code system (or “EHDAA”) used to regulate school funding. Codes are provided for the following categories for which schools receive additional government funding: moderate to severe behavioral disorders, intellectual disabilities, motor disabilities, speech and language disabilities, visual impairments, hearing impairments, autism spectrum disorder, psychiatric disorders, other atypical disorders. Conversely, the provincial system does not provide codes for students with mild or moderate learning difficulties (most prominently LD and ADHD), for which schools do not receive additional funding and for whom record-keeping (i.e., coding systems) and distribution of funds take place at the school level. Thus, students with LD and/or ADHD are identified as having a disability within administrative data but lumped in a residual category without being attributed a code for a specific disability. The variable *learning-related disability status* (0 = *no disability*, 1 = *disability*) was created by triangulating these variables to identify students with an IEP without an otherwise identified handicap or disability (who were excluded from the study). In other words, this variable identified students presenting mild to moderate disabilities affecting learning, that is, likely a LD and/or ADHD in almost all cases (*n* = 315).

#### Educational Attainment—High School Diploma and College Enrollment Since Leaving High School

Educational attainment data was determined through administrative government data compiled in 2022, which was available for the full analytical sample. High school graduation was indicated by a binary variable identifying participants who had earned a high school diploma or equivalent by 2022. College enrollment was measured with a binary variable identifying those who had enrolled in college at least once by 2022, although the exact timing of their enrollment could not be determined. The data set did not include enough participants having completed vocational training to examine this outcome in the analyses. Similarly, too few participants had completed a collegial degree or progressed to university-level studies to include these outcomes in the analyses.

#### Employment and Post-Secondary Outcomes at the Time of the Interview

Employment data was obtained through the Phase 2 interview data wherein participants declared current educational enrollment and employment and thus reflects participants’ situations at the time of the second interview. Data was available for the portion of the analytical sample who participated in the follow-up interviews (*n* = 386) and for whom employment data was collected (*n* = 362). Participants were asked to describe their current employment (e.g., hours, wage) and whether they considered it a “real” job corresponding to their career aspirations or a “fill-in” while waiting for something else (e.g., better conditions, different field). Responses were coded into two categories: temporary employment and career-related employment (for additional information, see Thouin et al., 2023). Participants were considered in employment only when employment was their main occupation; in other words, participants whose main occupation was education (i.e., reported being in full-time studies), regardless of if working part- or full-time, were not considered as employed either in a “fill-in job” or a “career job.” Participants neither in education nor employment were placed in a third category: Not in Education, Employment or Training (NEET). Altogether, these categories were used as employment outcomes as follows: (a) NEET (0 = *no*, 1 = *yes*), (b) main occupation—employed in temporary job (0 = *no*, 1 = *yes*), and (c) main occupation—employed in career-related job (0 = *no*, 1 = *yes*).

#### Sociodemographic Control Variables

Sociodemographic data was collected at T1 and includes age (in years) and control variables: gender (0 = *female*, 1 = *male*), visible minority (i.e., non-White) status (0 = *no*, 1 = *yes*), at least one parent born outside of Canada (0 = *no*, 1= *yes*), living with both parents/parents not separated (0 = *yes*, 1= *no*), mother full-time employment status (0 = *no*, 1 = *yes*), father full-time employment status (0 = *no*, 1 = *yes*), school location (0 = *rural*, 1 = *urban*), and SES measured by the highest parental education of either parent (0 = *high school or less*, 1 = *post-secondary*).

### Analyses

Analyses were conducted using IBM Statistics Package for Social Sciences Software (SPSS 26.0). Five hierarchal multiple logistic regressions were conducted to assess whether learning-related disability status predicted (a) High school diploma obtained, (b) College enrollment, (c) Being NEET, (d) Main occupation—Temporary employment, and (e) Main occupation—Employment in desired field. Each regression analyses included two models with two blocks each. Model 1 assessed whether and to what extent the presence of learning-related disability status explained each outcome after controlling for sociodemographics. Model 2 incorporated the interaction between learning-related disability status and gender to assess whether gender moderated the relationship between learning-related disability status and each outcome. Supplemental analyses were also conducted to investigate potential interactions between learning-related disability status and other control variables. As detailed above, participants with disability codes indicating non-learning disabilities (*n* = 11) or missing data for educational outcomes informed by administrative records (*n* = 21) were removed from the analyses. The employment model was estimated on a subset of 362 participants for whom interview data for employment outcomes were available. That is, participants lost to attrition in Phase 2 interviews (*n* = 127) and those having not reported employment (*n* = 24) were excluded from the analyses.

## Results

### Descriptive Statistics

Descriptive statistics for the overall analytical sample (*n* = 513) and relative to learning-related disability status (*n* = 315) are shown in Table 1. About half of the participants were male, from urban areas, had intact family structure, and had one parent with post-secondary education. About a third of the sample had a parent born outside of Canada or belonged to a visible minority, and about two thirds reported their father or mother employed full-time. Slightly more than half of the participants belonged in the learning-related disability status and those within this category are similar in distribution to the overall sample.

### Multiple Regression Models

Results predicting education and employment outcomes after controlling for sociodemographic variables as a function of both learning-related disability status and gender are shown in Table 2. Results for regression analyses indicating significant learning-related disability status by gender interactions are shown in Table 3. Control variable parameters, available upon request, are omitted for concision.

**Table 2.** Hierarchal Multiple Regression Analysis Predicting Education and Employment Outcomes in the Early 20s.

Outcomes		Parameters associated with learning-related disability status					Parameters associated with gender (male)				
		β	*SE*	*OR*	95% CI	*p*	β	*SE*	*OR*	95% CI	*p*
High school diploma	(*n* = 513)	–1.01	(0.20)	0.37	[0.25 0.55]	<.001	–0.42	(0.20)	0.66	[0.45 0.97]	.034
College enrollment	(*n* = 513)	–0.93	(0.22)	0.40	[0.26 0.61]	<.001	–0.68	(0.22)	0.51	[0.33 0.78]	.002
NEET	(*n* = 362)	1.00	(0.35)	2.72	[1.36 5.44]	.005	–0.64	(0.33)	0.53	[0.28 1.00]	.051
Employed—temporary job	(*n* = 362)	–0.08	(0.24)	0.92	[0.58 1.47]	.732	0.06	(0.23)	1.06	[0.68 1.67]	.801
Employed—career-related job	(*n* = 362)	0.182	(0.30)	1.20	[0.66 2.18]	.550	0.62	(0.30)	1.85	[1.03 3.33]	.040

*Note*. All models control for family structure, immigration and visible minority status, parental education, parental work status, and location (urban/rural). Full models available upon request. β = beta; *OR* = odds ratio; CI = confidence interval; NEET = neither in education, employment, or training.

**Table 3.** Hierarchal Multiple Regression Analysis Predicting Education and Employment Outcomes in the Early 20s With Learning-Related Disability Status × Gender Interactions.

	Parameters associated with gender, learning-related disability, and gender × disability interactions				
Predictor variables	β	*SE*	*OR*	95% CI	*p*
**Model predicting college enrollment**					
Gender (male)	–1.21	(0.33)	0.30	[0.16 0.58]	<.001
Learning-related disability status	–1.38	(0.31)	0.25	[0.14 0.46]	<.001
Learning-related disability status × gender (male)	0.95	(0.44)	2.59	[1.09 6.16]	.031
**Model predicting NEET status**					
Gender (male)	0.59	(0.58)	1.81	[0.59 5.59]	.303
Learning-related disability status	1.68	(0.48)	5.35	[2.11 13.56]	<.001
Learning-related disability status × gender (male)	–1.74	(0.70)	0.18	[0.05 0.69]	.013

*Note*. All models control for family structure, immigration and visible minority status, parental education, parental work status, and location (urban/rural). Full models available upon request. β = beta; *OR* = odds ratio; CI = confidence interval; NEET = neither in education, employment, or training. Results are shown only for outcomes with significant learning-related disability status by gender interactions.

#### Disability Status and Gender

Results presented in Table 2 show that after controlling for key background characteristics, participants with learning-related disabilities were significantly less likely to have a high school diploma or enroll in college and more likely to be NEET. For all these associations, the effect sizes as captured by odds ratio are non-negligible (*OR* = 0.37, 0.40, and 2.72, respectively). However, the likelihood of employment in a temporary job or career-related job did not significantly differ between participants with and without learning-related disabilities. As for gender, males had lower levels of educational attainment and engagement than females, as they were less likely to have a high school diploma and to enroll in college, again with non-negligible effect sizes (*OR* = 0.66 and 0.51, respectively). They had advantages in the employment sphere, however, as they were more likely to be employed in a career-related job (*OR* = 1.85), and marginally less likely to be NEET (*OR* = 0.53). Gender did not predict the likelihood of belonging in temporary employment (*B* = .06; *SE* = 0.23; *p* = .801).

#### Gender by Learning-Related Disability Interactions

**Figure 1.**
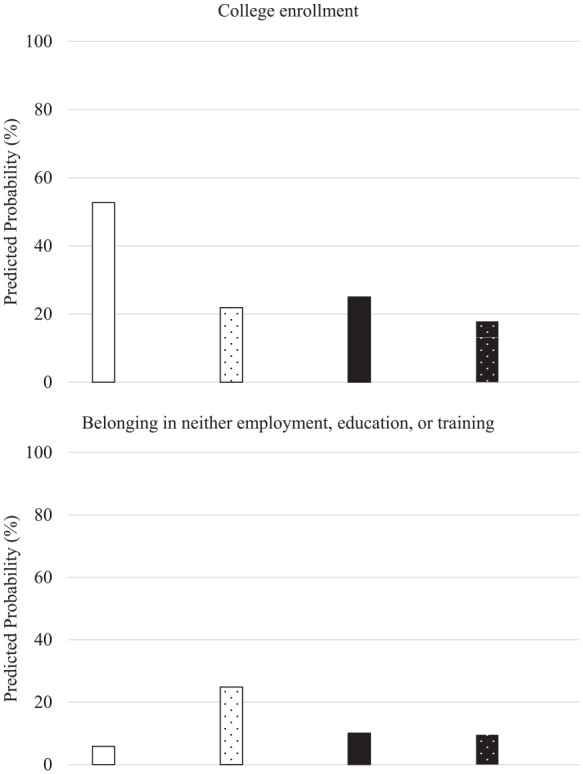
College Enrollment and Youth in Neither Employment, Education, or Training by Learning-Related Disability and Gender. *Note*. White bars: Girls. Black bars: Boys. Textured Bars: Learning-related disability.

As shown in Table 3, significant interactions between learning-related disabilities and gender emerged for college enrollment and NEET status. The initial model explained 17.5% (χ^2^ = 17.93, *p* < .001) of the variance in college enrollment. Adding the learning-related disability and gender interaction term as a predictor increased the explained variance to 18.3% (Δ*R* = 0.08, χ^2^ = 4.75, *p* = .029). Regarding the NEET status, the initial model explained 7.9% (χ^2^ = 8.74, *p* = .003) of the variance in the likelihood of belonging in NEET. The addition of the interaction term increased the explained variance to 9.4% (Δ*R* = 0.15, χ^2^ = 6.13, *p* = .013). To facilitate interpretation, predicted probabilities for these two outcomes were calculated for males and females with and without learning-related disabilities, while holding other variables constant (at their mean levels). As shown in Figure 1, girls without learning-related disabilities were most likely to be enrolled in college, followed by boys without learning-related disabilities, girls with learning-related disabilities, and boys with learning-related disabilities, respectively. Girls with learning-related disabilities were almost four times less likely to be enrolled in college versus those without; conversely, college enrollment probabilities between girls with learning-related disabilities and boys without were similar.

Also shown in Figure 1, girls with learning-related disabilities had the highest probability of belonging in NEET (approximately three times more likely than all the other groups), whereas girls without had the least. Likelihoods of belonging in NEET for boys with and without learning-related disabilities were similar.

There was no significant interaction between learning-related disabilities and gender in the model predicting high school attainment (*B* = 0.07; *SE* = 0.40; *p* = .854), and females in this group did not differ in their likelihood of obtaining a high school diploma beyond the general gender differences observed in the full sample. Likewise, the interactions between gender and learning-related disabilities were non-significant for temporary employment (*B* = −0.10; *SE* = 0.47; *p* = .835) and employment in a desired field (*B* = −0.66; *SE* = 0.60; *p* = .273).

### Supplemental Analyses

Supplemental analyses investigating interactions between learning-related disability status and other sociodemographic variables (minority status, immigration status, family structure, mother full-time employment status, father full-time employment status, school location, and SES) were also conducted. No significant interactions emerged (full results available upon request).

## Discussion

The purpose of this study was to better understand how high-incidence learning-related disabilities (mostly LD and ADHD) interact with gender to shape educational and employment outcomes during the school-to-work transition. Our findings contribute to the growing body of literature surrounding this issue demonstrating that youth with learning-related disabilities face challenges as they leave compulsory schooling. Specifically, youth with learning-related disabilities were less likely to obtain a high school diploma or enroll in college. More importantly, our findings also suggest that these young men and women face different obstacles when transitioning into education or employment, wherein girls specifically are more likely to be in neither.

### Impact of Learning-Related Disabilities on Education and Employment

Results concerning learning-related disabilities and educational attainment are consistent with current literature and provide additional evidence for the challenges faced both at the secondary and post-secondary levels; specifically, youth identified with learning-related disabilities in high school were less likely to obtain a high school diploma or enroll in college (Chatoor, 2021; Homsy & Savard, 2018). Although the overall enrollment in post-secondary education within this vulnerable population is increasing, youth with learning-related disabilities have yet to catch up to their normative peers. While research regarding the impact of these disabilities on employment is less exhaustive, our findings are consistent with claims that these youth may struggle to both enter and remain in the workforce (Arim, 2017; Chatoor, 2021; Rajah et al., 2023). Their enrollment in post-secondary education, albeit much more dampened than their peers, did not translate into higher chances of employment in either a temporary or career-related job. In other words, while much of the current literature considers rapid entry into the workforce as the go-to alternative for youth with learning-related disabilities who do not pursue education, they do not appear to enter the job market at higher rates than those without disabilities. As a result, they are overrepresented in NEET and at considerable risk for long-term social, financial, psychological, and health-related difficulties and lower quality of life (Davidson & Arim, 2019).

Contrary to existing literature, those who *did* manage to integrate into the job market, even if rapidly and without higher education, did not differ in temporary or career-related employment. This suggests that, while the transition into employment may be difficult, youth with learning-related disabilities may enter satisfactory employment equally as their peers once they have successfully entered the workforce and bypass some of the challenges traditionally observed in this population (Arim, 2017; Lansford et al., 2016). Alternatively, the fact that participants did not differ in the employment type may reflect the low educational attainment of the overall sample, wherein only 44% had obtained a high school diploma (35% vs. 60% in disability and non-disability groups, respectively). Although higher academic credentials would typically lead to better employment, the fact that so many participants among the non-disability group had dropped out of high school may have “leveled” the playing field, so that participants found themselves truncated to temporary or unsatisfying jobs by way of limited opportunities regardless of disability.

Nonetheless, these findings present a strong argument for the inclusion of transition planning while youth are identifiable and accessible during compulsory schooling (see Etscheidt et al., 2023). Currently, IEPs in Canada do not uniformly include transition planning and as such, many youths with high-incidence disabilities who exit compulsory schooling not only lose access to important supports but are left with little direction toward other services (Towle, 2015). While those who continue in education (and to a lesser extent, employment) have access to some degree of service infrastructure, evidence shows that most students with disabilities still do not disclose once they enter post-secondary education for a variety of reasons, including ambiguity in availability, eligibility, and utility, and fear of discrimination (Lindsay, Cagliostro, Albarico, et al., 2018; Stamp, 2020). Similarly, youth who do not pursue post-secondary education or employment find themselves without readily available infrastructure to guide their daily activities (Towle, 2015). Transition planning appears critical not only in facilitating integration into either post-secondary schooling or employment but also in increasing literacy around service access and use, especially for those at risk of falling through the cracks of being neither in education nor employment (Arim, 2017; Towle, 2015).

### Impact of Gender on Education and Employment

Results regarding gender on educational attainment are consistent with existing literature in that boys are generally underrepresented at all levels of education in North America (Morris et al., 2018; Schimmele et al., 2021). Although boys were less likely to complete high school or enroll in college, they were marginally less likely to find themselves unemployed when not in education and more likely to be employed in a career-related field. Together, these findings nuance the relationship between gender and educational and employment outcomes and suggest that rapid entry into the workforce is likely easier for young men than it is for women for a variety of reasons (Hirano et al., 2023; Lindsay et al., 2018). Notably, young men appear to have access to a broader range of vocational options that do not require post-secondary credentials but still provide stable and well-paid employment, whereas women likely find themselves restricted to low-wage jobs in service or childcare fields (Hirano et al., 2023; Lindsay et al., 2018). In fact, recent findings suggest that young men most often forego post-secondary education by personal choice or because they simply do not need it to attain their career objectives; conversely, their female counterparts likely pursue further education by necessity and as a means of access to equivalent work conditions (Lindstrom et al., 2012; Parker, 2021).

### Gender Differences in Education and Employment in Youth With Learning-Related Disabilities

Our results provide preliminary evidence for disparities between young men and women with learning-related disabilities in both education *and* employment. Specifically, the presence of learning-related disabilities appeared to almost close the post-secondary enrollment gap between boys and girls observed in the general population, wherein girls have the advantage. Research has shown that girls with learning or attentional difficulties are at a disadvantage when it comes to identification and service provision during compulsory schooling (Auer et al., 2023; Little & McLennan, 2010; Mowlem et al., 2019). Although they may persevere into higher education, findings here suggest that they do so at a significantly lesser degree than female peers without such difficulties, perhaps due to accumulated academic difficulties and lack of, or delay in, support provisions (Burenkova et al., 2021; Lindstrom et al., 2020). This identification bias renders learning or attentional disabilities comparatively rare among girls, and so the presence of a diagnostic label may impact young girls’ self-perception and academic expectations (e.g., teachers, parents) differently than for boys, whose difficulties are more normalized. If so, a learning-related disability diagnosis, like learning disability (LD) or ADHD, could contribute to gradual academic disengagement and decreased aspirations, particularly among young girls with such disabilities (Burenkova et al., 2021; Lindstrom et al., 2020) and potentially explain the disparity in post-secondary enrollment observed in this study.

Although girls with learning-related disabilities were less likely to enroll in college, they were not more likely to transition into employment either; instead, they rather belonged to neither category. Although to be interpreted with caution, this may suggest that girls who do not continue in education, perhaps due to their lower expectancy of academic success, may attempt to enter employment only to face the disadvantages in the workforce. As a result, young girls with learning-related disabilities leaving compulsory schooling may face a double disadvantage—in education and employment—and be at higher risk of falling in the gap *between* these two pathways, leaving them especially vulnerable to additional adverse outcomes (Davidson & Arim, 2019).

Another interpretation is that young women with such disabilities who do not opt for education pursue avenues outside employment. For example, a recent study on Canadian youth NEET found that approximately a third reported caring for family as their main occupation, and amongst these, more than 90% were women (Davidson & Arim, 2019). It may be that, for women with learning-related disabilities, aspirations and trajectories may deviate away from education or employment and toward alternative trajectories that seem more promising.

### Strengths and Limitations

This study offers important insight into youth with high-incidence learning-related disabilities’ trajectories. First, whereas most research investigates education or employment exclusively, our study considers both in parallel and allows for a comprehensive outlook on youth’s transitional outcomes. Second, while most relevant research focuses on student or post-graduate populations using a single temporal point of reference, our longitudinal approach allows the inclusion of individuals with such disabilities regardless of if they have left education or transitioned into employment without following normative educational pathways. Third, this methodology allowed the inclusion of additional sociodemographic variables (e.g., parental education) that may also be important in shaping the school-to-work transition. Finally, while many relevant studies (especially based in Canada) rely on retrospective self-reporting of disabilities, our methodology based on administrative data during compulsory schooling increases the reliability of our identification measure.

Nonetheless, limitations should be recognized. First, the recruitment strategy was designed to oversample youth likely to face various difficulties (e.g., having dropped out, attending public schools in low SES neighborhoods). As a result, the sample had a relatively high school dropout rate (40%) in comparison to general trends across Canada and the United States (Homsy & Savard, 2018; Statistics Canada, 2019). Nevertheless, the relative difference in high school diplomacy between students with and without learning-related disabilities is in line with disparities in attainment generally observed in youth with LD and/or ADHD, and findings may speak to the cumulated impact of learning-related disabilities in addition to other sociodemographic risk factors (Arim, 2017; LDAO, 2024; Prince et al., 2019). With regard to sociodemographic background characteristics, the analyses controlled for key indicators measured in adolescence, while the participants were still in compulsory education. However, there are other important decisions shaping young adults’ social realities that come into play as youth enter adulthood, such as parenthood, marital status, and educational attainment, most prominently whether they obtain a high school diploma or equivalent. Future research incorporating a mediation analysis should look closely at these factors as processes involved in youth with learning-related disabilities’ life trajectories.

Second, though our sample included youth with learning-related disabilities regardless of whether they pursue further education or not, too few students entered or progressed into several types of post-secondary education (e.g., vocational training or adult education, university) to be considered as important educational outcomes in our analyses. Third, this study relies on the assumption that students transition from compulsory schooling to either education, employment, or neither, but these trajectories are not exhaustive or exclusive. Moreover, these outcomes did not consider potential subgroups within those in NEET. For example, while overall consequences of belonging in NEET are well-documented, NEET youth whose main occupation is caring for children or who are actively searching for employment appear at lesser risk for certain difficulties in comparison to those doing neither (Davidson & Arim, 2019). While preliminary analyses made possible by the interview data indicate that most NEET youth in the present sample did wish to return to education or seek employment and had not begun raising a family, research considering comparative individual contexts is necessary to better understand the realities of youth in NEET by lack of opportunity versus by choice (Davidson & Arim, 2019).

Finally, while the inclusion of administrative records allowed us to bypass problems innate to self-identification and attrition over time for certain outcomes, the information available in administrative records was limited. Without the variable directly identifying students with LD or ADHD, learning-related disabilities could only be deduced by considering that students with an IEP and no otherwise indicated non-learning-related disability (categories for which were nonetheless exhaustive and comprehensive) likely fell within this category. Likewise, the exclusion of other disabilities so to increase our measurement’s reliability meant the exclusion of students with complex profiles who may have an LD and/or ADHD alongside other high-incidence disabilities impacting academic performance (e.g., anxiety; Fletcher & Grigorenko, 2017). While efforts are being made to improve service provision for these vulnerable students, inability to properly record-keep and identify these students limits the ability to inform these efforts (Larose et al., 2022; Murray et al., 2021; Prince et al., 2019). Quality and reliability of enrollment data may even be lower for vocational versus mainstream programs, which is problematic seeing that students with disabilities may opt for such programs as alternatives to general post-secondary studies. Nevertheless, the present study offers an example of what can be accomplished by matching survey data with administrative records, and as such supports efforts to facilitate the transmission of information between government and researchers.

### Practice Implications and Future Research

This study sheds light on the continued challenges that youth with learning-related disabilities face during and after compulsory schooling, as even with the implementation of an IEP, many continue to face disadvantages in education *and* employment once they leave high school. Our findings speak to the intersectionality of disability and other sociodemographic factors, such as gender, in predicting educational and employment outcomes (Haber et al., 2016; Mazzotti et al., 2021). Implications for practice are twofold.

First, in line with prominent frameworks highlighting the need to consider multiple contextual layers to understand the transition out of compulsory schooling for young people with disabilities (Trainor et al., 2020), the findings underscore the joint relevance of sociodemographic and contextual predictors of post-secondary and subsequent employment success in orienting service provision. Alongside learning-related disability, considering individual characteristics like gender may be helpful in flagging students most in need and understanding said needs within the context of individual circumstances. Besides individual characteristics like gender, there are other well-documented and emerging factors, such as parental involvement and self-realization, that could also be leveraged to provide tailored supports to those in need (Mazzotti et al., 2021).

Second, findings highlight the added value of considering education and employment outcomes simultaneously, albeit separately. Previous findings suggest that predictors of post-secondary education and employment outcomes vary in their ability to predict educational versus employment success, and that strategies to improve one may even sometimes hinder the other (Haber et al., 2016; Monahan et al., 2011). For example, high involvement or motivation in employment may undermine school participation, and vice versa (Monahan et al., 2011). Thus, services and transition planning for youth during and after compulsory schooling should not only consider sociodemographic factors, as described above, but also youths’ specific post-school goals (Haber et al., 2016; Mazzotti et al., 2021).

Future studies should continue to investigate the relationship between such disabilities in high school and early-adulthood outcomes using longitudinal methods, while considering a wide array of outcomes other than simply in-versus-out of schooling or employment (e.g., by considering the type of educational program, career field). As demonstrated here, there is value in considering a more reliable and targeted definition of learning-related disabilities, even more so LD specifically, beyond self-report or general disability data to improve accuracy in our understanding of this population’s specific needs. Thus, we hope that our study speaks to the importance of improving means of access to administrative data so to bridge the gap between research and institutional practices. Finally, we underline the importance of service provision, including transition planning, that considers not just disability but other important individual factors, such as gender, so to ensure equitable access to quality education and employment for all young men and women.
